# OncoSplicing: an updated database for clinically relevant alternative splicing in 33 human cancers

**DOI:** 10.1093/nar/gkab851

**Published:** 2021-09-23

**Authors:** Yangjun Zhang, Xiangyang Yao, Hui Zhou, Xiaoliang Wu, Jianbo Tian, Jin Zeng, Libin Yan, Chen Duan, Haoran Liu, Heng Li, Ke Chen, Zhiquan Hu, Zhangqun Ye, Hua Xu

**Affiliations:** Department of Urology, Tongji Hospital, Tongji Medical College, Huazhong University of Science and Technology, Wuhan 430030, China; Institute of Urology of Hubei Province, Wuhan 430030, China; Department of Urology, Tongji Hospital, Tongji Medical College, Huazhong University of Science and Technology, Wuhan 430030, China; Institute of Urology of Hubei Province, Wuhan 430030, China; Department of Urology, Tongji Hospital, Tongji Medical College, Huazhong University of Science and Technology, Wuhan 430030, China; Institute of Urology of Hubei Province, Wuhan 430030, China; Department of Urology, Tongji Hospital, Tongji Medical College, Huazhong University of Science and Technology, Wuhan 430030, China; Institute of Urology of Hubei Province, Wuhan 430030, China; Department of Epidemiology and Biostatistics, School of Public Health, Tongji Medical College, Huazhong University of Science and Technology, Wuhan 430030, China; Department of Urology, The First Affiliated Hospital of Nanchang University, Nanchang 330000, China; Department of Urology, The First Affiliated Hospital, School of Medicine, Zhejiang University, Hangzhou 310000, China; Department of Urology, Tongji Hospital, Tongji Medical College, Huazhong University of Science and Technology, Wuhan 430030, China; Institute of Urology of Hubei Province, Wuhan 430030, China; Department of Urology, The Second Affiliated Hospital of Kunming Medical University, Kunming 650000, China; Department of Urology, Tongji Hospital, Tongji Medical College, Huazhong University of Science and Technology, Wuhan 430030, China; Institute of Urology of Hubei Province, Wuhan 430030, China; Department of Urology, Tongji Hospital, Tongji Medical College, Huazhong University of Science and Technology, Wuhan 430030, China; Institute of Urology of Hubei Province, Wuhan 430030, China; Department of Urology, Tongji Hospital, Tongji Medical College, Huazhong University of Science and Technology, Wuhan 430030, China; Institute of Urology of Hubei Province, Wuhan 430030, China; Department of Urology, Tongji Hospital, Tongji Medical College, Huazhong University of Science and Technology, Wuhan 430030, China; Institute of Urology of Hubei Province, Wuhan 430030, China; Institute of Urology of Hubei Province, Wuhan 430030, China; Cancer Precision Diagnosis and Treatment and Translational Medicine Hubei Engineering Research Center, Wuhan 430030, China; Department of Biological Repositories, Zhongnan Hospital of Wuhan University, Wuhan 430030, China; Department of Urology, Zhongnan Hospital of Wuhan University, Wuhan 430030, China

## Abstract

Alternative splicing (AS) represents a crucial method in mRNA level to regulate gene expression and contributes to the protein complexity. Abnormal splicing has been reported to play roles in several diseases, including cancers. We developed the OncoSplicing database for visualization of survival-associated and differential alternative splicing in 2019. Here, we provide an updated version of OncoSplicing for an integrative view of clinically relevant alternative splicing based on 122 423 AS events across 33 cancers in the TCGA SpliceSeq project and 238 558 AS events across 32 cancers in the TCGA SplAdder project. The new version of the database contains several useful features, such as annotation of alternative splicing-associated transcripts, survival analysis based on median and optimal cut-offs, differential analysis between TCGA tumour samples and adjacent normal samples or GTEx normal samples, pan-cancer views of alternative splicing, splicing differences and results of Cox’PH regression, identification of clinical indicator-relevant and cancer-specific splicing events, and downloadable splicing data in the SplAdder project. Overall, the substantially updated version of OncoSplicing (www.oncosplicing.com) is a user-friendly and registration-free database for browsing and searching clinically relevant alternative splicing in human cancers.

## INTRODUCTION

Alternative splicing (AS) represents a crucial method in regulating gene expression and plays roles in development, adaptation and many other physiological processes (1). Alternative splicing of a protein-coding gene may result in decaying transcript isoforms due to mRNA instability or in novel protein isoforms with counterbalanced functions (2). Irregular alternative splicing of a disease-related gene not only inhibits its original role but also produces functional effects in the occurrence and development of many diseases, including cancers (3,4).

Recently, the increasing accessibility of next-generation sequence (NGS) data has facilitated further interpretation of RNA-seq data at the splicing level and diversified the exploration of algorithms that are used to detect AS events. For example, Ryan *et al.* explored SpliceSeq software to analyse RNA-seq data and then implemented it in the TCGA pan-cancer cohort, finding hundreds of thousands of AS events across more than ten thousand samples (5,6). Similarly, Kahles et al. explored SplAdder software to analyse alternative splicing and then implemented it in the TCGA pan-cancer and GTEx cohorts, resulting in a large number of AS events (7,8). The SplAdder software identify AS events by constructing an augmented splicing graph, which can be added with detected novel exon or intron. Although applied to the same TCGA RNA-seq data, these two software produced considerably different landscapes of alternative splicing in TCGA cancers, especially for novel AS events and multiple skipping exons. Moreover, these two projects are different in splicing quantification: SpliceSeq software takes all read counts covered in the splicing exon as AS-associated reads, while SplAdder takes into consideration only the read counts covered in the splice junctions. These differences in AS detection and quantification enriched the AS landscape in TCGA cancers. Besides, in the SpliceSeq database, exon indexes were organized by the reconstructed nonredundant locations, which made it very easy to understand the AS event but difficult to match it to the annotated transcripts. For the SplAdder project, splicing data and raw count data were stored integrally for all cancer types but separately for different splice types, in hdf5 file format, which makes data hard to reuse.

To systematically study the clinical effect of alternative splicing in cancers, we developed the OncoSplicing database for visualization of survival-associated and differential AS events based on PSI (percent spliced in) data from the TCGA SpliceSeq database, as described in our previous paper (9). Here, we will introduce our updated database for an integrative view of clinically relevant alternative splicing in TCGA cancers and GTEx tissues based on all splicing data in the TCGA SpliceSeq database and raw count data in the SplAdder project. The PSI values of AS events in the SplAdder project were regenerated by implementing a modified pipeline to the raw count data. Several new features were added to the current version of the database, including annotation of AS-associated transcripts, survival analysis of dichotomized PSI values based on an optimal cut-off, comparison analysis between TCGA tumour samples and GTEx normal samples, pan-cancer views of PSI distribution, differences and results of Cox’PH regression, identification of clinical indicator-relevant and cancer-specific AS events and downloadable PSI data for TCGA cancers and GTEx tissues in the SplAdder project.

Other helpful databases about alternative splicing in humans have recently been developed, such as CancerSplicingQTL (10), ASCOT (11), ExonSkipDB (12) and RJunBase (13). CancerSplicingQTL (http://www.cancersplicingqtl-hust.com/#/) is an sQTL resource that provides useful tools for identifying potential SNPs controlling alternative splicing in human cancers. ASCOT (http://ascot.cs.jhu.edu/) provides analysis and visualization of alternative splicing and gene expression across tens of thousands of RNA-seq data in the public archive using annotation-free methods. ExonSkipDB (https://ccsm.uth.edu/ExonSkipDB/) integrates detailed information of exon skipping events and their associations with mutation and methylation based on multi-omic evidence in the TCGA SplAdder project. RJunBase (http://www.rjunbase.org/) focuses on identification of RNA splice junctions and provides visualization of junction-level expression profiles in cancer and non-cancer tissues. However, these databases provide neither clinically relevant analysis of alternative splicing in cancers nor determinate relationships between alternative splicing and annotated transcripts. Hence, OncoSplicing (www.oncosplicing.com) is still a unique database that provides analysis of clinically relevant alternative splicing based on a large number of AS events across TCGA cancer and GTEx tissue samples. We believe that this database could be a convenient tool to link alternative splicing to clinical features and annotated transcript isoforms and may be very useful in the research community.

## DATA COLLECTION AND PROCESSING

### Splicing data from the SpliceSeq project

**Figure 1.**
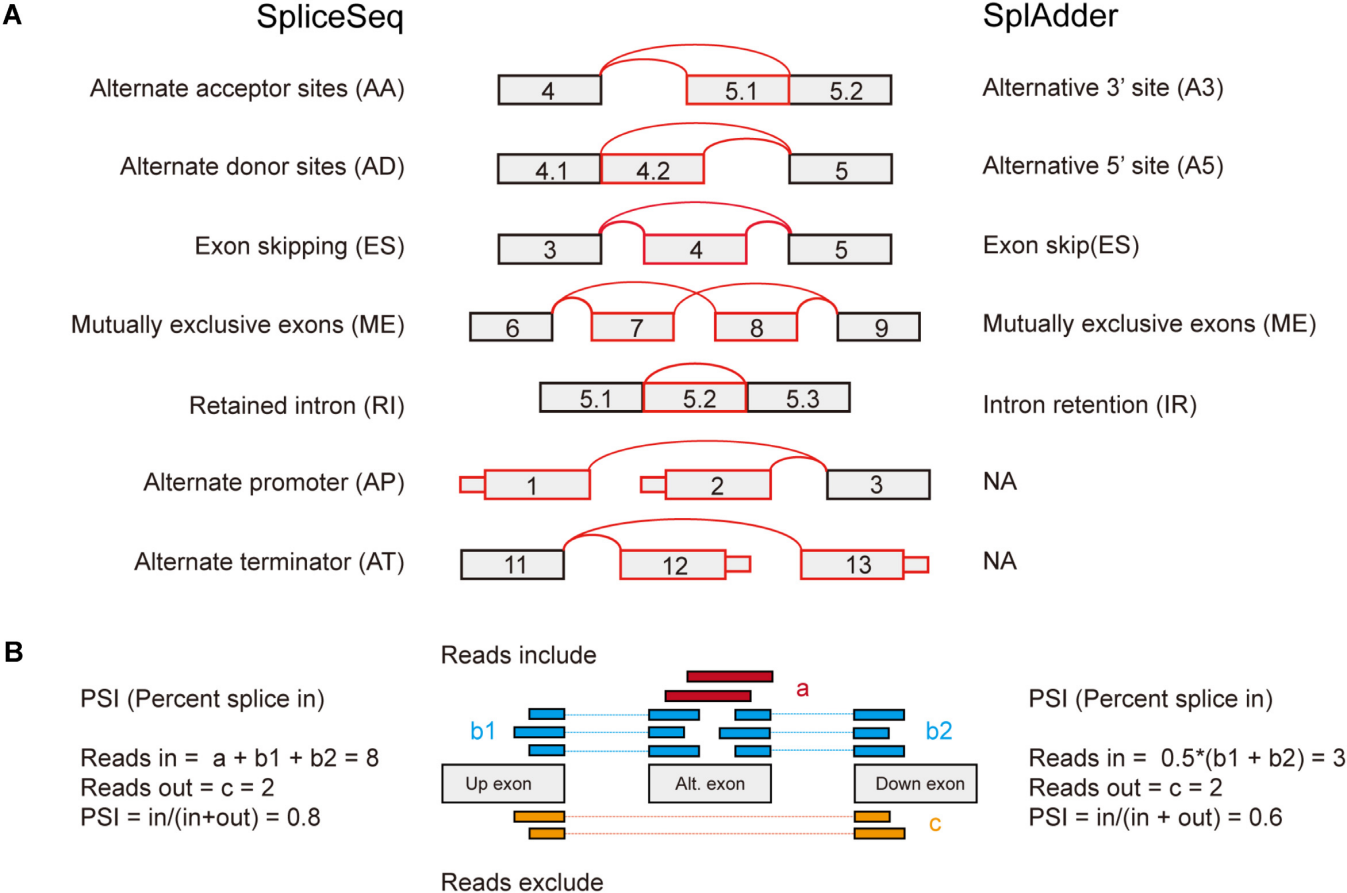
Splice types and quantification method in the SpliceSeq and SplAdder project. (**A**) Splice model for splice types detected in the SpliceSeq and the SplAdder projects. Exon number was illustrated in the box and splice junctions and alternatively spliced exon were indicated in red lines and boxes respectively. (**B**) Diagram of alternative splicing detection and PSI calculation by the SpliceSeq and SplAdder software. The PSI-calculation method of intron retention in both SpliceSeq and SplAdder is similar to that of exon skipping described for SpliceSeq software, except that the alternate exon is replaced by retained intron.

**Figure 2.**
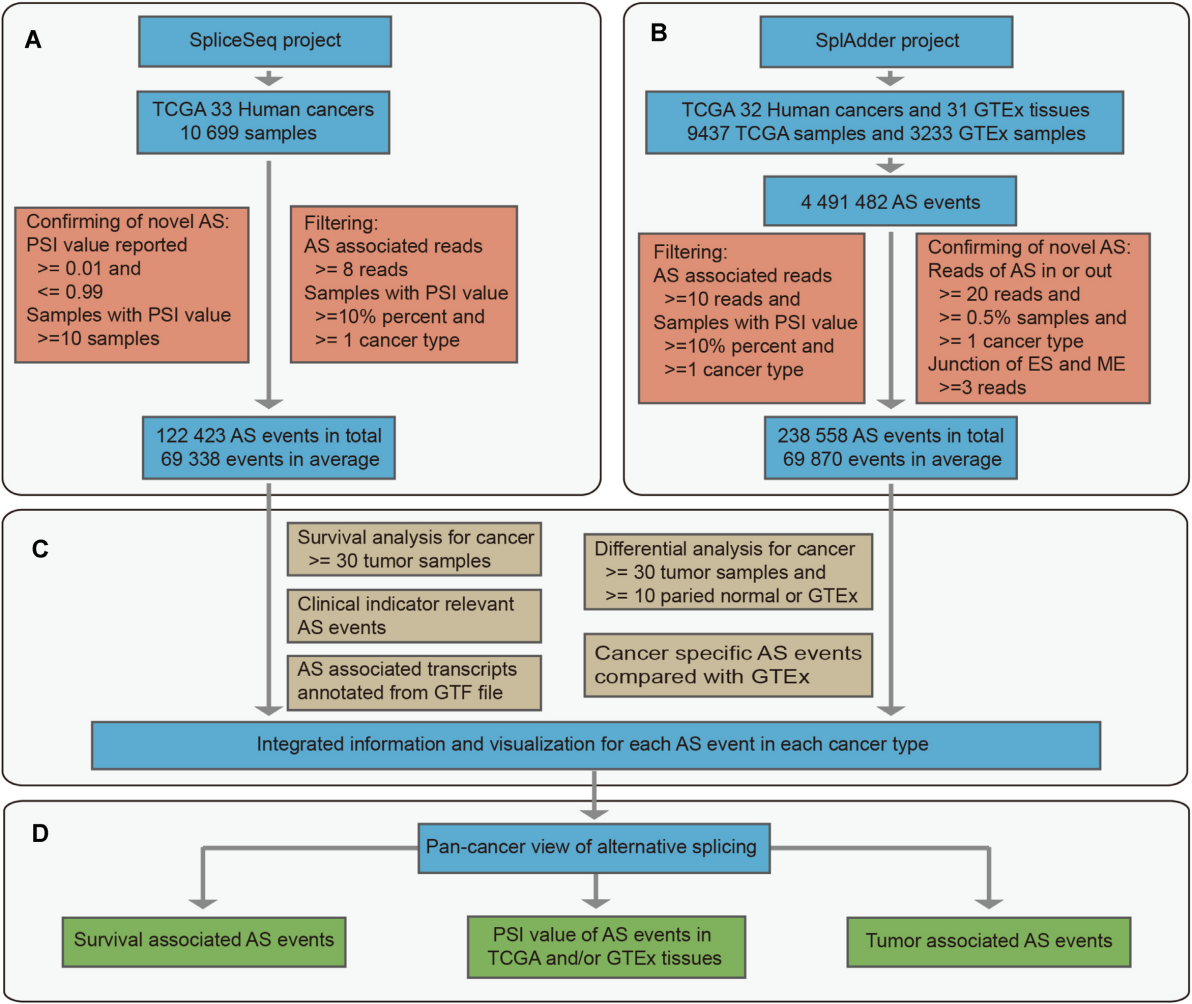
Data processing and database construction pipeline. (**A**) Splicing data collection and processing in the SpliceSeq project. (**B**) Data collection and processing in the SplAdder project. (**C**) Analyses for alternative splicing associated to survival, sample type, cancer specificity, clinical indicators and annotated transcripts. (**D**) Pan-cancer analyses and views for PSI distribution, survival association and sample types difference of alternative splicing.

**Figure 3.**
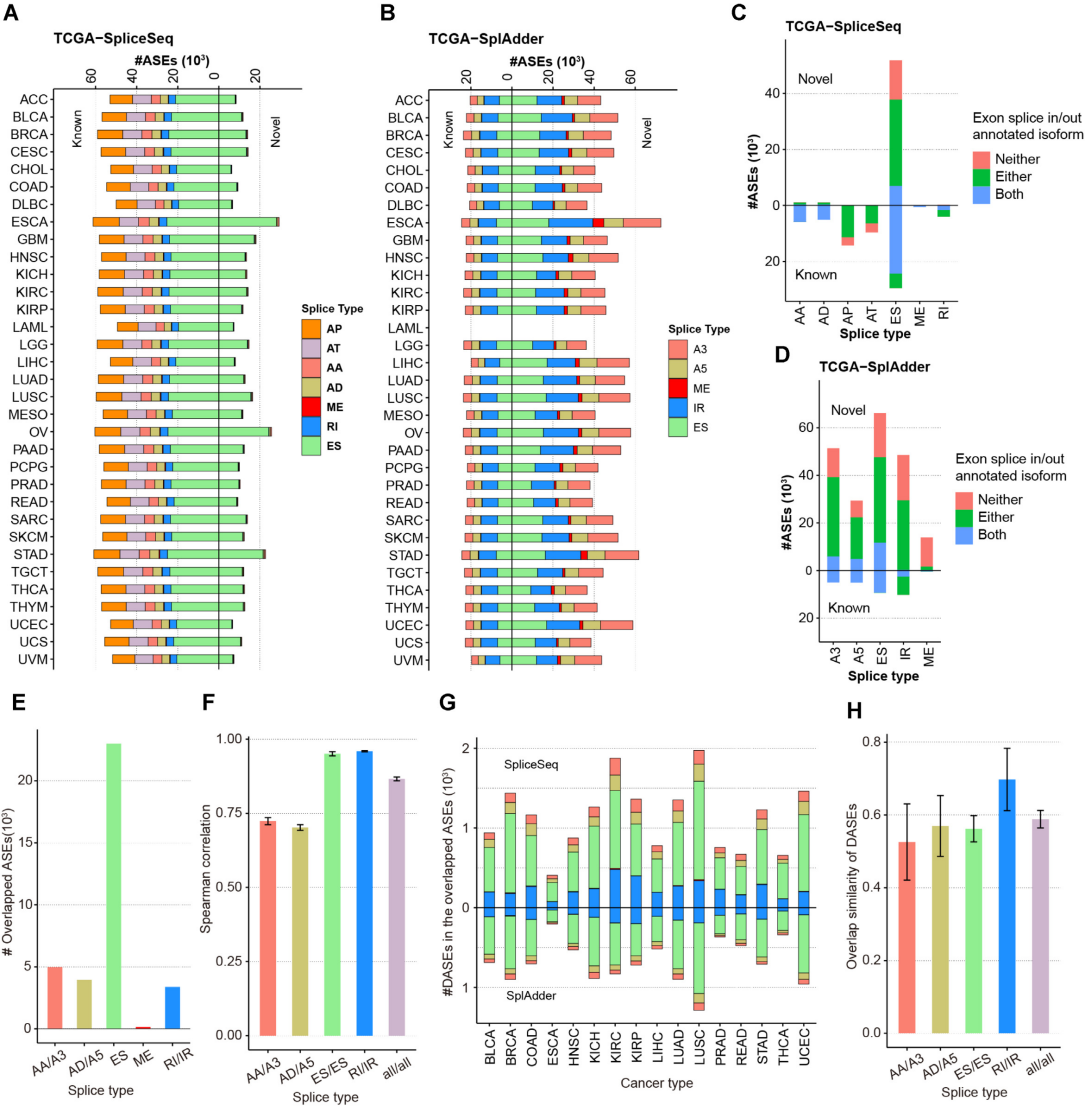
The landscape of alternative splicing in OncoSplicing. (**A**) Statistics of alternative splicing of seven splice types in the SpliceSeq project. The full names of splice types and cancer types were listed respectively in the Figure 1A and the Supplementary Table S1. (**B**) Statistics of alternative splicing of five splice types in the SplAdder project. The cancer type LAML was not included in this project. (**C**) Statistics of AS associated transcripts in the SpliceSeq project. Blue, green and red bar indicate respectively the number of AS events that both, either and neither of exons splice in and splice out that are associated with at least one annotated transcript. (**D**) Statistics of AS associated transcripts in the SplAdder project. (**E**) The number of overlapped AS events in five splice types detected in both the SpliceSeq and SplAdder projects. (**F**) Consistency of AS events detected in the two projects. Spearman correlation analyses were performed by median PSI values of the overlapped AS events across each cancer type. For each splice type, error bar indicate mean plus or minus standard deviation of correlation coefficients of different cancer types. (**G**) The number of DASEs detected from the overlapped AS events in the SpliceSeq and SplAdder projects. The five colours in each cancer type indicate different splice types described in (B). (**H**) Overlap similarity of DASEs detected in these two project. Overlap similarity is defined as ratio that equal to the intersection size of two sets divided by the minimum size. For each splice type, error bar indicate mean plus or minus standard deviation of overlap similarity of different cancer types. ASEs: alternative splicing events. DASEs: Differential alternative splicing events.

PSI values of all AS events for 33 cancer types were downloaded from the TCGA SpliceSeq database (http://projects.insilico.us.com/TCGASpliceSeq/PSIdownload.jsp) with parameter percent-samples-with-values set as no less than 10%. All seven splice types were collected in OncoSplicing, including alternative acceptor sites (AA), alternative donor sites (AD), exon skipping (ES), mutually exclusive exons (ME), retained intron (RI), alternate promoter (AP) and alternate terminator (AT) (Figure 1A). AS-associated reads were defined as reads supporting exon splice in (reads-in), and reads supporting exon splice out (reads-out). The PSI value generated by the reads-in divided by the sum of reads-in and the reads-out (Figure 1B) is a common index to quantify different uses of alternative exons. In the SpliceSeq project, the PSI values of AS events were calculated only if AS-associated reads were no less than 8; otherwise, they were assigned empty values. Moreover, only AS events with PSI values in more than 10% of samples in at least one cancer type were included in this database (Figure 2). As a result, there were 122 423 AS events detected in total and 69 338 AS events detected per cancer type in average (Figure 3A).

### Splicing data from the SplAdder project

Raw count data were downloaded from the GDC data portal (https://gdc.cancer.gov/about-data/publications/PanCanAtlas-Splicing-2018). All five splice types in this project were collected in OncoSplicing, including alternative 3′ site (A3), alternative 5′ site (A5), exon skip (ES), mutually exclusive exons (ME) and intron retention (IR) (Figure 1A), which were respectively same as AA, AD, ES, ME and RI in the SpliceSeq project. Raw count data were extracted for each splice type to quantify reads-in and reads-out. We modified the PSI calculation in the SplAdder project by normalizing the read quantification by the number of splice junctions (14), which might to some extent affect PSI quantification of the ME and ES splice types (Figure 1B). AS events were confirmed and kept for further analysis only if they met one of the following criteria: (i) known AS events or (ii) novel AS events with reads-in and reads-out minima of 20 in at least 0.5% of samples in at least one cancer type, and a minimum of reads for different junctions of no less than 3 for the ES and ME splice types (15,16). Finally, AS events were filtered by the following criterion: AS-associated reads >10 in at least 10% of samples in at least one cancer type (Figure 2). As a result, there were 238,558 AS events detected in total and 69,870 AS events detected per cancer type in average (Figure 3B).

### Collection of phenotype data

From the UCSC Xena database (https://xenabrowser.net/datapages/), phenotype data for TCGA and GTEx samples were separately downloaded in the GDC pan-cancer cohort and GTEx cohort (17). Survival data including overall survival (OS), progression free interval (PFI), disease free interval (DFI) and disease specific survival (DSS) were downloaded in each TCGA cancer cohort respectively from the UCSC Xena database (18). From the EBI database (https://www.ebi.ac.uk/ena/browser/view/PRJNA75899), we obtained the run accession and sample title for each experiment in the GTEx project, which linked the sample title in the SplAdder project to the GTEx phenotype (19).

### Annotation of AS-associated transcripts

An AS-associated transcript means that the transcript contained splice junctions in the AS event, associated with either exon splice in or splice out. From the genome annotation file (GRCh37/GENCODEv19), exons were organized from 5′ to 3′ for each annotated transcript. The gene structure file that defined the chromosome position of every single exon in the SpliceSeq project was downloaded from the TCGA SpliceSeq database (https://bioinformatics.mdanderson.org/TCGASpliceSeq/TCGA_SpliceSeq_Gene_Structure.zip). Splice junctions for each AS event were identified by the locations of splice exons and upstream and downstream exons. For the SplAdder project, splice junctions in each AS event were identified from the event regions. These AS-associated splice junctions were then mapped to structuralized transcripts to identify AS-associated transcripts for the SpliceSeq and SplAdder projects. As a result, 101 877 (83.2%) AS events in the SpliceSeq project and 169 426 (71%) AS events in the SplAdder project were annotated with at least one transcript (Figure 3C and D).

### Survival analysis

For each AS event, PSI values were divided into two groups by a median cut-off and an optimal cut-off in each cancer type. The optimal cut-off was predicted using survival data by the ‘surv_cutpoint’ function in the R package ‘survminer’. Dichotomy univariate Cox’PH regression analyses were performed for AS events with PSI values in >30 samples using the R package ‘survival’. AS events with log-rank *P*-values <0.05 based on the median cut-off were defined as survival-associated alternative splicing events (SASEs). Survival analysis of AS events based on the optimal cut-off was only presented in Kaplan–Meier visualization (‘KMplot’ function). Survival analyses were implemented for OS, PFI, DFI and DSS survival data respectively and for AS event only if it with effective sample size >30, survival event >5 and minima of group size >10. Significant SASEs identified by each survival data were integrated as clinically relevant splicing events on the ‘ClinicalAS’ page and the statistic results can be found in Supplemental Table S1.

### Differential analysis

Differential AS analyses were performed for cancer types with at least 30 TCGA tumours and 10 adjacent normal or paired GTEx samples in either the SpliceSeq or SplAdder project. The Wilcoxon rank-sum test was used to evaluate the significance of differences between tumour and normal tissues. Splicing events with an absolute delta PSI >0.1 and a Benjamini–Hochberg (BH) adjusted *P*-value <0.05 were defined as significant differential alternative splicing events (DASEs). Comparison analyses for AS events between TCGA tumours and paired GTEx tissues in the SplAdder project were only presented in the visualization (‘TNplot’ function). Significant DASEs were also integrated as clinical indicator (sample type) relevant splicing events on the ‘ClinicalAS’ page and the statistic results can be found in Supplemental Table S1.

### Identification of AS events relevant to clinical indicators

Basic patient information, including age, sex and race, and nonredundant and variant clinical indicators, was manually collected and separated into two groups for each cancer type. Clinical indicators in a cancer type were reserved for further analysis only if there were >20 records per group. Differential AS analysis was performed between two groups for each indicator, and only AS events with a delta PSI greater than 0.1 and a BH adjusted *P*-value <0.05 were considered significant clinically relevant AS events.

### Identification of cancer-specific AS events

In the SplAdder project, we assume that cancer-specific AS events alternatively splice only in TCGA cancers but not in GTEx normal tissues (tissue type ‘Cells’ were excluded). AS events were considered cancer-specific AS only if they met one of the following criteria: (i) PSI >0.99 in >90% of GTEx samples and <0.95 in >10% of tumour samples for at least one TCGA cancer type or (ii) PSI <0.01 in >90% of GTEx samples and >0.05 in >10% of tumour samples for at least one TCGA cancer type.

## DATABASE CONTENT

### Samples in OncoSplicing

In total, OncoSplicing contains 10 699 TCGA samples (9950 tumour and 749 adjacent normal samples) across 33 cancer types in the SpliceSeq project and 9437 TCGA samples (8744 tumour and 693 adjacent normal samples) across 32 cancer types and 3233 GTEx samples across 31 tissue types in the SplAdder project. After quality control, the SplAdder project filtered some of the TCGA samples, including all LAML samples. Cancer and tissue names and their sample size in each project is detailed in the summary table (Supplementary Table S1).

### AS events in OncoSplicing

After confirming novel AS events and filtering by samples with PSI values, a total of 122 423 (53 990 novel and 68 433 known) AS events across 33 cancer types and a range of 56 729 to 90 561 AS events in different cancer types were identified in the SpliceSeq project (Figure 3A). For the SplAdder project, there were 238 558 (209 017 novel and 29 541 known) AS events in total, and a range of 57 163 to 97 054 AS events were identified in different cancer types (Figure 3B). By match the event regions of alternative splicing, there were 35 402 overlapped AS events detected in both these two projects (Figure 3E). Spearman correlation analyses for PSI values of the shared AS events showed significant consistency between these two projects, especially for splice type exon skipping and intron retention (Figure 3F). For the overlapped events, though the number of DASEs identified in the SpliceSeq were about 1.65-fold more than that in the SplAdder project, the overlap similarity in average were over 0.5 in all splice types (Figure 3G and H). In addition, there were a plenty of AS events detected different in these two projects. In the SpliceSeq, for example, there were 40 541 (49.9%) multiple exons skipping events included in the splice type exon skipping, which the SplAdder project did not detect. Novel AS events were mostly detected in exon skipping in the SpliceSeq project, while were equivalently detected in all splice types in the SplAdder project (Figure 3A and B). The size of AS events for each cancer type in each project is detailed in the summary table (Supplementary Table S1).

## DATABASE ORGANIZATION AND WEB INTERFACE

The OncoSplicing website was based on the springboot and layui frameworks and was deployed under a centOS 7.3 linux system. To make it convenient and efficient for data querying, we organized our data in OncoSplicing by using MySQL supported by Aliyun RDS (Relational Database Service). Several R codes were used to implement data visualization. The OncoSplicing database provides a user-friendly web interface and facilitates searching, browsing and downloading splicing data for TCGA cancers and GTEx tissues. It can be freely available online (www.oncosplicing.com) and has been tested on Safari, Firefox and Chrome browsers, for desktops and tablet PCs or mobile phones.

**Figure 4.**
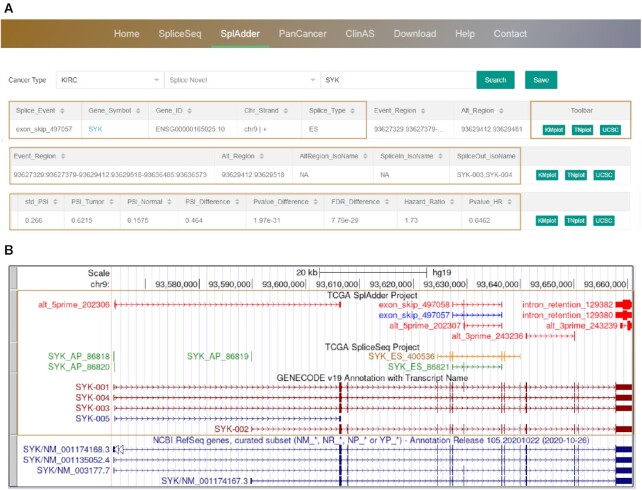
Overview of the OncoSplicing database. (**A**) Browser bar in OncoSplicing and an example of search results on the ‘SplAdder’ page. After querying gene ‘SYK’ in cancer type ‘KIRC’, a result table responses with detailed information of the query gene, alternative splicing, statistic results and function buttons. (**B**) An example of customized tracks in the UCSC genome browser for the query gene, presenting explanatory diagrams of alternative splicing events and annotated structure of transcripts in the SplAdder and SpliceSeq projects.

Results table that responses to searches for AS events are characterized by diverse information, including splice event (structured by gene symbol, splice type and splice ID), gene information (including gene symbol, Ensembl gene ID, chromosome and strand), splice regions (alternate exons, upstream exon and downstream exon for SpliceSeq, or event regions and alternate region for SplAdder), splice type, novel splice, overlapped event, AS associated transcript (including alternate exon number in isoforms, isoforms with exon splice in and isoforms with exon splice out), clinical indicator (clinical indicator types and subgroups) and statistical results (including sample percent with PSI values, standard deviation of PSI values, median PSI in tumour, median PSI in normal, PSI difference, p-value of difference, FDR of difference, hazard ratio, PSI cut-off and log-rank p-value of hazard ratio) (Figure 4A). If AS events exist, all results will be returned in a table and can be saved by clicking on the ‘Save’ button; otherwise, a message appears with ‘Sorry, your query does not exist in this result’. The ‘Splice Novel’ box can be used to furtherly filter novel and known AS events. By clicking on the gene name embedded with a hyperlink, users are directed to the Ensembl database (20). In the button region, by clicking on the UCSC button, users are directed to the UCSC genome browser (21), which is characterized by customized tracks with annotated transcript structures, and explanatory diagrams of detected AS events in the SplAdder and SpliceSeq projects (Figure 4B). In addition, the statistical results for AS events in the two projects are shown on the ‘Home’ page. A short summary detailing the meaning of PSI, the construction of the database, the functions provided by the database and the sample size of each project is available on the ‘Help’ page. All data in OncoSplicing can be downloaded on the ‘Download’ page. Users are welcome to provide feedback on any related issues by email on the ‘Contact’ page.

### Queries on the ‘SpliceSeq’ and ‘SplAdder’ pages

On the ‘SpliceSeq’ or ‘SplAdder’ pages, to query alternative splicing in a TCGA cancer type, users can select the cancer type from the pull-down list, enter a gene symbol or splice event in the search box and click on the ‘Enter’ key or the ‘Search’ button to obtain the query AS events. By clicking on the ‘KMplot’ button, survival curves in the pop-up window will display the relationship between patient survival and dichotomized PSI values based on the median cut-off and/or optimal cut-off. By clicking on the ‘TNplot’ button, boxplots will display the PSI difference or PSI distribution for cancer types with or without paired adjacent normal or GTEx samples. These pop-up vector diagrams can be downloaded in pdf format by clicking on the ‘Download’ button.

### Queries on the ‘PanCancer’ and ‘ClinicalAS’ pages

On the ‘PanCancer’ page, users can query alternative splicing in a pan-cancer view by selecting a project type (‘SpliceSeq’ or ‘SplAdder’) from the pull-down list. Similarly, by entering a gene symbol or splice event in the search box and clicking on the ‘Enter’ key or the ‘Search’ button, relative AS information and plotting buttons are returned. Users can click on the ‘PanPlot’ button to generate a vector boxplot diagram displaying PSI distributions in all TCGA cancer types and/or GTEx tissues. The ‘PanDiff’ button provides a pan-cancer view of the queried AS event (detected in at least 3 cancers) for PSI differences between tumour samples and adjacent normal and/or GTEx normal samples. The ‘PanOS’ and ‘PanPFI’ button provides a pan-cancer view of the queried AS event (detected in at least three cancers) for Cox’PH results of OS and PFI data respectively, based on the median PSI cut-off and/or predicted optimal PSI cut-off. For the results table on the ‘PanCancer’ page, SASEs, DASEs and cancer-specific AS were annotated in the ‘SurvivalOS_AS’, ‘SurvivalPFI_AS’, ‘Differential_AS’ and ‘CancerSpecific_AS’ columns, respectively.

On the ‘ClinicalAS’ page, all significant clinically relevant AS events were integrated, including AS events related to clinical indicators, sample types (boxplot) and survival. After choosing a project, users can select an existing cancer type and/or a clinical indicator to browse significantly relevant AS events or input a gene symbol to search for alternative splicing of the query gene in different subgroups of clinical indicators. The ‘CIplot’ provides a visualization for PSI or survival differences of a selected AS event between two subgroups of a clinical indicator.

## SUMMARY AND FUTURE DIRECTIONS

Although most AS events in OncoSplicing belong to either the SpliceSeq or SplAdder project, there were still a large number of AS events detected in these two projects at the same time. For example, exon skipping of the seventh exon of SYK, a well-known AS event, was detected in both the SpliceSeq (SYK_ES_86821) and SplAdder (exon_skip_497057) projects and was validated in our previous study (22). While VHL is commonly known as a gene with alternative splicing of the second exon, which is annotated in the Ensembl database (20,22). A novel exon of VHL was only detected by the SplAdder project (exon_skip_371642), which is annotated in the NCBI RefSeq but not in the Ensembl database (23). Hence, the combinational use of these data in OncoSplicing may either ensure the confidence of alternative splicing for genes in a specific condition or enlarge our knowledge in alternative splicing or transcript structure for genes in rare conditions. Overall, OncoSplicing is an informative resource that provides a user-friendly interface to browse or search for AS events in 33 TCGA cancers and 31 GTEx tissues in the SpliceSeq or SplAdder projects. Millions of vector diagrams and dozens of datasets are provided for download, scientific usage and further integrative studies.

The accessibility of next-generation sequences and accurate software have led to rapid increases in research related to alternative splicing. We will continue to maintain and update OncoSplicing in the future by including more analyses of phenotype-related alternative splicing, splicing regulatory correlations and AS-associated transcripts annotated by long-read sequencing to maintain OncoSplicing as a useful and up-to-date resource for the research community. This database will be a convenient tool and an important resource for researchers studying alternative splicing in cancer.

## DATA AVAILABILITY

R code used to implement visualization in the database is publicly available on GitHub: https://github.com/yjzhang2013/OncoSplicing/.

## Supplementary Material

gkab851_Supplemental_Files
