# Supplementary material for: OncoSplicing: an updated database for clinically relevant alternative splicing in 33 human cancers
Source: Nucleic Acids Res. 2021 Sep 23;50(D1):D1340–7. doi: 10.1093/nar/gkab851 (PMC8728274; doi:10.1093/nar/gkab851)
Supplement: gkab851_Supplemental_Files [file gkab851_supplemental_files.zip › Supplementary_table_1_legend.docx]

**Table Legend**

Supplementary table 1: Statistical results of sample size, detected AS events, survival analyses and differential analysis for each cancer type included in the SpliceSeq and the SplAdder project. The cancer type LAML was excluded in the SplAdder porject. The statistical results of cancer types that lack effective clinical data are assigned a value of "NA".
